# *Au courant* computation of the PDB to audit diffraction anisotropy of soluble and membrane proteins

**DOI:** 10.1016/j.dib.2018.05.072

**Published:** 2018-05-19

**Authors:** Xavier Robert, Josiane Kassis-Sahyoun, Nicoletta Ceres, Juliette Martin, Michael R. Sawaya, Randy J. Read, Patrice Gouet, Pierre Falson, Vincent Chaptal

**Affiliations:** a Molecular Microbiology and Structural Biochemistry institute, UMR5086 CNRS Univ-Lyon, F-69367 Cedex 7, Lyon, France; b Howard Hughes Medical Institute, University of California, Los Angeles, CA 90095, USA; c Molecular Biology Institute, Department of Chemistry and Biochemistry, University of California, Los Angeles, CA 90095, USA; d Department of Haematology, University of Cambridge, Welcome Trust/MRC Building, Hills Road, Cambridge CB2 0XY, England, UK

**Keywords:** X-ray diffraction, Diffraction anisotropy, Membrane proteins, Macromolecule crystals

## Abstract

This data article makes available the informed computation of the whole Protein Data Bank (PDB) to investigate diffraction anisotropy on a large scale and to perform statistics. This data has been investigated in detail in “X-ray diffraction reveals the intrinsic difference in the physical properties of membrane and soluble proteins” [1]. Diffraction anisotropy is traditionally associated with absence of contacts in-between macromolecules within the crystals in a given direction of space. There are however many case that do not follow this empirical rule. To investigate and sort out this discrepancy, we computed diffraction anisotropy for every entry of the PDB, and put them in context of relevant metrics to compare X-ray diffraction in reciprocal space to the crystal packing in real space. These metrics were either extracted from PDB files when available (resolution, space groups, cell parameters, solvent content), or calculated using standard procedures (anisotropy, crystal contacts, presence of ligands). More specifically, we separated entries to compare soluble *vs* membrane proteins, and further separated the later in subcategories according to their insertion in the membrane, function, or type of crystallization (Type I *vs* Type II crystal packing). This informed database is being made available to investigators in the raw and curated formats that can be re-used for further downstream studies. This dataset is useful to test ideas and to ascertain hypothesis based on statistical analysis.

**Specifications Table**

**Table:** 

Subject area	*Biology*
More specific subject area	*Crystallography*
Type of data	*Excel sheet document*
How data was acquired	*Advanced computation on Protein Data Bank**[2]**data*
Data format	*Raw and curated*
Experimental factors	*Each Protein Data Bank entry were retrieved for both experimental diffraction data and deposited model, and further processed and classified according to biologically driven criterion.*
Experimental features	*Separation between soluble and membrane proteins; membrane proteins were further separated in different subclasses. For each entry, diffraction anisotropy was calculated and compared to many parameters to investigate the cause of the phenomenon.*
Data source location	*All entries were retrieved from the Protein Data Bank**[2]*.
Data accessibility	*The data is made available as supplemental information of this article*

**Value of the data**

• First broad analysis of the spread of diffraction anisotropy across the entire Protein Data Bank.
• Allows researchers to compare their anisotropy to all other available entries and better gage their data.
• These data set ground to challenge established ideas and to further investigate diffraction data from macromolecule crystals.

## 1 Data

In these data, X-ray diffraction anisotropy is calculated for each entry of the Protein Data Bank and put into perspective with relevant structural information, such as solvent content, resolution, crystal contacts, space group, presence of ligands, etc., to investigate correlations. The aim of these data is to investigate differences between soluble and membrane proteins, so these two types of proteins were identified and separated. Membrane proteins were further separated into subclasses according to their insertion in the membrane, fold, or function to link differences with biology.

## 2 Experimental design, materials and methods

### 2.1 Data mining and computation

As of February 24th, 2016, a local copy of the RCSB Protein Data Bank (PDB) was made including all the deposited structures in PDB formatted coordinate files as well as all the crystallographic structure factors in mmCIF format. To this date, out of 115,888 available structures, 103,530 were solved by X-ray crystallography and 92,995 related structure factors files were accessible. For further processing, these last were converted from mmCIF to CCP4 MTZ format with the sf-convert software version 1.204 (developed at RCSB and downloadable at http://deposit.pdb.org/software). By this mean, 92,930 structure factors files were successfully converted, while 65 were not due to various file format issues. We then developed an automated Linux script in Bash programming language that sequentially performed the following tasks for each of the PDB/MTZ couple of files obtained previously:

– Several data were directly extracted from the PDB file header: the most recent deposition/revision year (PDB ‘REVDAT’ record), the resolution in angstrom (‘REMARK 2 RESOLUTION’ record), the space group and the unit cell parameters (‘CRYST1’ record), the data collection temperature (‘REMARK 200 TEMPERATURE’ record), all compounds/ligands sorted by their 3-letters hetID codes (‘HETNAM’ records), as well as the list of terms relevant to the entry (‘KEYWDS’ record).
– In addition to these keywords and for each membrane proteins entries, we extracted the name of the protein from the ‘Membrane proteins of known 3D structure’ database (http://blanco.biomol.uci.edu/mpstruc/) leaded by S.H. White (University of California, Irvine).
– When available, the solvent content (in percent) was also retrieved (‘REMARK 280 SOLVENT CONTENT’ record). If not, it was calculated using the program matthews_coef from the CCP4 software suite version 7.0 [3].
– When applicable, the percentages of four classes of protein secondary structure elements (helices, strands, turns and coils) were calculated using the program mkdssp version 2.2.7 included in CCP4.
– A crystal contacts ratio value was determined by dividing the number of crystal contacts in the unit-cell (computed using ncont from CCP4 with a maximum distance cutoff of 4.0 Å) by the total number of atoms, including heteroatoms and solvent (i.e. all ‘ATOM’ and ‘HETATM’ PDB records).
– We employed the ‘UCLA Diffraction Anisotropy Server’ [4] script that we modified to take advantage of the last available revisions of CCP4. Thus, for each PDB entry, the anisotropic delta-B value was computed with Phaser [5] both using amplitude and intensity data, when available. Furthermore, the resolution limits at which F/σ(F) drops below 3.0 was determined using the program Truncate from CCP4, this for each of the 3 principle axes of the anisotropic ellipsoid. A ‘delta_res’ value was then deducted by subtracting the lowest resolution limit to the highest one. In addition, the Wilson B-factor was computed with Phaser using amplitude and intensity data, when available. A ratio between the previously calculated anisotropic delta-B value and this Wilson B-factor was then deducted, both with amplitude and intensity data when available. Finally, the total number of reflections was extracted from the structure factors file as well as the number of reflections that were rejected during the anisotropy correction cycles performed by Phaser, this allowing us to determine the percentage of rejected reflection during this process.

Thus, from the starting set constituted by 92,930 entries, we were able to compute 92,218 aniso_b based on amplitude data, 26,319 based on intensities and 92,154 delta_res values. The differences came from the fact that a number of structure factor files did not contain intensity data and/or accurate information (i.e. missing or null σ(F), σ(I) values, etc.).

All these data were joined, sorted by PDB entry code and imported in an Excel 2013 (Microsoft Corporation) spreadsheet.

### 2.2 Curation

For reasons described in [6] and the behavior of anisotropy over the years (Fig S6D in ref [6]), we decided to only retain structures obtained after 2005 in order to compare entries of similar difficulty levels and susceptible to have comparable anisotropic behavior. Also, in order to compare reasonably well-behaved structures, only data diffracting to less than or equal to 5 Å resolution were kept, and anisotropic delta-B values on amplitudes above 150 Å^2^ were rejected. In addition, all crystal contacts ratio over 1 were removed from the analysis. Thus, our final dataset consisted of 76,458 entries with 74,928 and 1411 calculated anisotropic delta-B values on amplitudes (soluble and membrane proteins, respectively); and 23,125 and 487 values on intensities.

### 2.3 Subsets extraction

From this curated database, 13 subsets were then extracted based on distinct structural or biological criteria. These last derived from the classification provided by the ‘Membrane proteins of known 3D structure’ database (http://blanco.biomol.uci.edu/mpstruc/). These subsets are: soluble proteins; membrane proteins; membrane proteins structures solved in detergents, lipidic cubic phase (extracted as described by M. Caffrey [7]) or bicelles; α-helical or β-barrel transmembrane proteins; monotopic membrane proteins; membrane ATPase, electron-transfer, channel, receptor and transporter proteins. Finally, two other subsets (embedded membrane proteins and proteins with extramembranous domains) were extracted based on visual inspection of their spatial arrangements information, visualized using the ‘Orientations of Proteins in Membranes’ (OPM) database [8].

### 2.4 Code availability

The present database is generated using an automated Linux Bash script we developed (tested on CentOS 7.x). This last is available with no restrictions upon request to the corresponding author. It can be executed on any Linux distribution as long as the CCP4 software suite version 7.0 (or superior) is installed. Moreover, a local copy of the PDB is also required: this includes all the deposited structures in PDB formatted coordinate files as well as all the crystallographic structure factors in mmCIF format to be converted in MTZ format. An additional Linux Bash script performing these file mirroring and conversion steps is available upon demand as well.
